# Protein-Protein Interactions of Viroporins in Coronaviruses and Paramyxoviruses: New Targets for Antivirals?

**DOI:** 10.3390/v7062750

**Published:** 2015-06-04

**Authors:** Jaume Torres, Wahyu Surya, Yan Li, Ding Xiang Liu

**Affiliations:** School of Biological Sciences, Nanyang Technological University, 60 Nanyang Drive, Singapore 637551, Singapore; E-Mails: Li.Yan@ntu.edu.sg (Y.L.); Wsurya@e.ntu.edu.sg (W.S.); dxliu@ntu.edu.sg (D.X.L.)

**Keywords:** coronavirus, envelope protein, respiratory syncytial virus, small hydrophobic protein, cytoplasmic helical domains, protein-protein interactions

## Abstract

Viroporins are members of a rapidly growing family of channel-forming small polypeptides found in viruses. The present review will be focused on recent structural and protein-protein interaction information involving two viroporins found in enveloped viruses that target the respiratory tract; (i) the envelope protein in coronaviruses and (ii) the small hydrophobic protein in paramyxoviruses. Deletion of these two viroporins leads to viral attenuation *in vivo*, whereas data from cell culture shows involvement in the regulation of stress and inflammation. The channel activity and structure of some representative members of these viroporins have been recently characterized in some detail. In addition, searches for protein-protein interactions using yeast-two hybrid techniques have shed light on possible functional roles for their exposed cytoplasmic domains. A deeper analysis of these interactions should not only provide a more complete overview of the multiple functions of these viroporins, but also suggest novel strategies that target protein-protein interactions as much needed antivirals. These should complement current efforts to block viroporin channel activity.

## 1. The Envelope (E) Protein in Coronaviruses (CoVs)

### 1.1. Coronaviruses

Coronaviruses (CoV) typically affect the respiratory tract and gut of mammals and birds. CoVs belong to the subfamily *Coronavirinae* in the family *Coronaviridae*, and are organized into four genera [1,2,3]: α, β, γ and δ. The first coronavirus was isolated in 1937 [4]—an avian infectious bronchitis virus (IBV) which until today can seriously devastate poultry stocks. Human coronaviruses (HCoV) were first identified and cultivated in the 1960s, from the nasal cavities of patients suffering from common cold [5]. Approximately 30% of common colds are caused by two human coronaviruses-OC43 and 229E. Of particular medical interest are the virus responsible for the severe acture respiratory syndrome (SARS), which produced a near pandemic in 2003 [6], and the recently emerged Middle East respiratory syndrome coronavirus (MERS-CoV), which after 3 years has caused hundreds of deaths [7].

Currently, no effective licensed treatments exist against coronavirus infection [8,9,10], although live vaccines consisting of attenuated viruses are a promising strategy [11,12], along with fusion inhibitors (reviewed in [13]). However, the possibility of reappearance of virulent phenotypes, drug side effects, and resistance calls for continued antiviral development. The latter depends on an intimate knowledge of the coronavirus molecular biology, described extensively elsewhere [14] and, increasingly, of their accessory proteins [15]. In this review, only the coronavirus envelope (E) proteins will be described in some detail.

### 1.2. General Features of the Envelope (E) Protein in CoVs

The envelope (E) proteins are short polypeptides (76–109 amino acids) and are encoded by a CoV subgenomic RNA either as a monocistronic or a polycistronic mRNA [14,16]. Most CoV E proteins are present at low concentrations in virions [17,18,19,20], with the exception of the E protein in IBV [21]. CoV E proteins are found abundantly in internal membranes [19,22,23,24]. For example, in MHV and SARS-CoV, E protein is found in the ER-Golgi intermediate compartment (ERGIC), where virions assemble [25,26].

**Figure 1:**
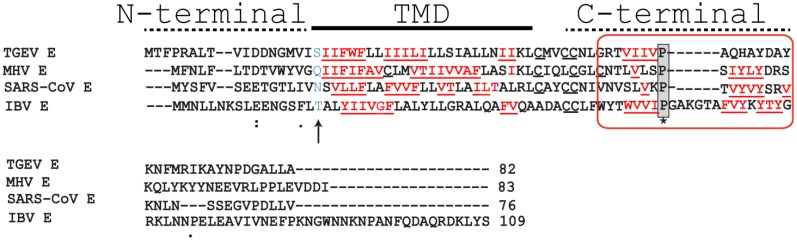
Alignment of representative CoV E proteins. Sequences of E proteins in α-, β- and γ-coronaviruses: TGEV (transmissible gastroenteritis virus) (α), MHV (murine hepatitis virus) and SARS (β), and IBV (γ), with TM domain (TMD) indicated. The fully conserved Proline in the C-terminal domain (highlighted grey) is flanked by predicted β-sheet structure at both sides, forming a hypothetical β-coil-β motif (BCBM) (red square). Polar residues that may be critical for channel activity are shown in blue (arrow). Helix-destabilizing residues in TMD and BCBM are highlighted red, bold and underlined.

Like other viroporins, and despite their small size, CoV E proteins have been found to be critical for pathogenesis. Earlier studies showed that over-expression of E protein from MHV and SARS-CoV induced apoptosis [27]. However, when cells were infected with a recombinant SARS-CoV lacking the E gene-a more biologically relevant system–it was found that this increased apoptosis in cell culture [28], whereas administration of E protein in trans reduced the stress response in cells infected with rSARS-CoV-ΔE. The same E protein anti-apoptotic effects were observed in cells subjected to other treatments that also elicited cell stress [28]. Deletion of E protein also reduced pathogenicity and mortality in animal models [29], and this has led to the development of live vaccines based on E-deleted or E-truncated virions [30,31,32]. Although the precise causes of attenuation are not known, they appear to be contributed by different parts of the E protein, which encompass the cytoplasmic C-terminal tail [33] as well as its transmembrane (TM) domain [34] (see Figure 1). In fact, in SARS-CoV E, the integrity of the TM domain and preservation of channel activity was shown to be important for inflammasome activation and elevated production of pro-inflammatory IL-1β. The latter effects were abolished by introduction of channel inactivating mutations N15A and V25F [34].

In IBV, E protein has been shown to play a role in the secretory pathway, altering lumenal environments and rearranging secretory organelles, and leading to efficient trafficking of virions [35,36,37,38,39]. These rearrangement of host cell membranes, e.g., the Golgi complex [37], are observed during CoV infections, and virions appear in large vacuoles derived from Golgi/ERGIC membranes [38]. The IBV E mutation T16A, aligned with channel-inactive mutation N15A in SARS-CoV E (Figure 1, arrow), resulted in decreased Golgi disruption [35,36], and this effect could not be restored by conservative polar substitutions Ser, Asn, or Gln [35].

In addition, a mutant IBV E where the TM domain was replaced with a non-oligomerizing TM domain of vesicular stomatitis virus glycoprotein (VSV G) was defective in release of infectious virus particles [36]. Particles appeared to accumulate intracellularly, pointing to the TM domain of IBV E as important for the forward trafficking of cargo to plasma membrane through the Golgi complex.

These effects could be due either to a loss of channel activity or the loss of TM domain integrity, with subsequent disruption of protein-protein interactions. Although channel activity of the T16A mutant that disrupted Golgi rearrangement was not measured [35], we have later found this mutant to be channel-inactive [40], suggesting a possible link between channel activity and Golgi rearrangement.

### 1.3. Structural Studies and Relevant Domains of CoV E Proteins

To understand viroporin biology, it is essential to complement cellular or *in vivo* studies with biophysical and structural studies on purified protein. Although structural data are not available for most CoV E proteins, SARS-CoV E [41,42,43,44,45,46], MERS E [47] and IBV E [40] have all been shown to have a single α-helical TM domain (Figure 1). This TM domain forms homopentameric channels with poor ion selectivity [48,49]. The topology of E protein channels has been a subject of controversy [35,50,51], but a recent study [26] of untagged SARS-CoV E protein in infected cells produced a model with cytoplasmic C-terminal domain and lumenal N-terminus, and this topology is also likely in other E proteins [20,24,26,35].

**Figure 2:**
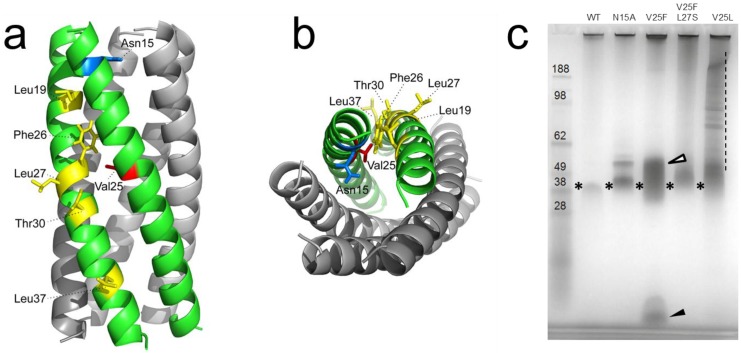
Model of pentameric TM α-helical bundle of SARS-CoV E and electrophoretic mobility of truncated E (8–65), E_TR_, TM mutants. (**a**) Model of the pentameric TM with residues which mutations resulted in channel inactivation, Val25 (red) and Asn15 (blue), and residues found changed in revertant mutants (yellow); adapted from [34]; (**b**) same as (**a**), in top view; (**c**) PFO-NuPAGE gel of E_TR_ and various mutants. The pentameric size of E_TR_ is approximately 45 kDa (*). Delayed bands in N15A and V25F (white arrow) and monomer (black arrow) are indicated. V25L also showed higher oligomers (dotted line).

The only structural data available for a CoV E protein is for SARS-CoV E, where the TM domain has been characterized in some detail in lipid membranes [42] and in DPC micelles [46]. In these models, Asn15 is facing the lumen of the channel [42] whereas Val25 is involved in helix-helix interactions with other subunits [46] (Figure 2a,b). Mutations at these residues abolished channel activity *in vitro* [52], and introduction of these mutations in a recombinant SARS-CoV resulted in *in vivo* attenuation in a mouse model [34]. Interestingly, revertant mutants that regained fitness and pathogenicity were recovered, and sequencing identified acquired mutations at the E protein TM domain. Those mutations that compensated for V25F clustered along the helix interface opposite to Val25 (Figure 2a,b). Synthetic peptides corresponding to the E protein TM domain bearing these “revertant” mutations, e.g., N15D, V25L, or V25F-L19A, regained channel activity as measured in black lipid membranes [34]. These revertant mutants also recovered pentameric integrity, as shown by an analysis of oligomeric size for mutants of a truncated form of SARS-CoV E protein, E_TR_ *i.e.*, E (8–65) (Figure 2c). In this assay, we used perfluorooctanoic acid polyacrylamide gel electrophoresis (PFO-NuPAGE), a system that preserves the native oligomeric size in membrane proteins [53,54]. Compared to the wild type E_TR_ (WT), both channel-inactive mutants N15A and V25F caused some migration changes of the E pentameric form (stars), showing delayed species above the pentamer band (see white arrow). Destabilization was obvious for V25F, where a monomeric species can be observed at the bottom of the gel (black arrow).

However, both V25F revertant mutants, V25F-L27S and V25L, exhibited a more compact pentamer than V25F, and no low molecular weight band corresponding to monomers (Figure 2c). This clearly shows that these mutants restored pentamer stability along with concomitant channel activity. In fact, the sedimentation equilibrium profile of these two revertant mutants, V25F-L27S and V25L, could be fitted to a monomer-pentamer model with log K_a_ = 16.7 and 17.5, respectively [40], comparable to the wild-type E_TR_ (log K_a_ = 16). Mutants N15A and V25F could not be examined by analytical ultracentrifugation because of their poor solubility. Overall, these data suggest that both channel activity and cytoplasmic tail integrity [33] are required simultaneously to achieve virulence.

**Figure 3:**
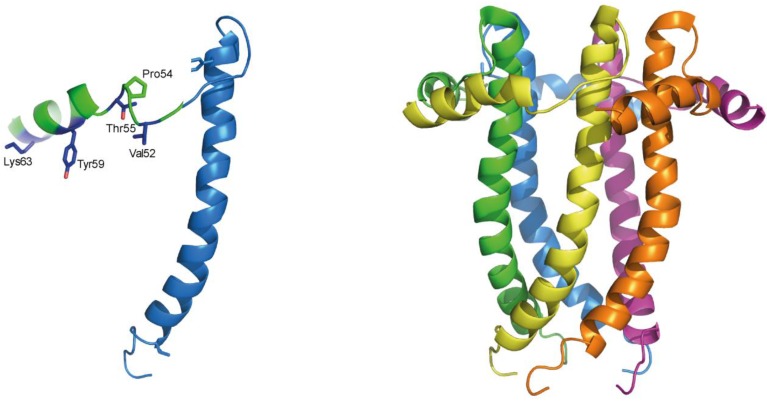
Structural model of SARS-CoV E. (**left**) Solution NMR-based model of E_TR_ (residues 8–65) in detergent micelles that shows that the predicted β-structure after the conserved proline residue (Pro54 to Lys63) is in fact α-helical; adapted from [45]; (**right**) pentameric model reconstructed from the E_TR_ monomer [45].

The C-terminal domain of E proteins contains a totally conserved proline residue, which in β- and γ-coronaviruses is at the center of a predicted β-coil-β motif, or BCBM (Figure 1). The construct E_TR_, E (8–65), which encompasses this motif, has been studied in mixed SDS/DPC micelles [45] (Figure 3). However, in that enviroment this motif was mostly α-helical (residues 54–65). In addition, the secondary structure of full length SARS-CoV E protein in lipid bilayers is almost completely α-helical and is not consistent with the presenc of β-sheet structure in that part of the protein [45]. Nevertheless, fragment 46–60 adopts 100% β-structure when studied as a synthetic peptide [44,55], and synthetic peptides inspired in that domain form amyloid fibers [55,56].

This discrepancy may point to the existence of a frustrated structure, poised for conformational change to β-structure. In SARS-CoV E, this region was found to redirect a plasma membrane protein to the Golgi region, and mutations designed to increase its α-helical propensity, V56A-Y57A-V58A-Y59A (“AAAA” mutant), disrupted membrane localization [57]. Indeed, purified E_TR_ bearing these same mutations produced an IR spectrum with slightly less β- and more α-structure [45]. However, although it is possible that a small fraction of the population has a BCBM folded as β-sheet structure, the reported cellular effects caused by the said “AAAA” mutant could have been equally caused by disruption of protein-protein interactions mediated by that exposed cytoplasmic domain.

The proposition of a “frustrated” BCBM is reminiscent of viral internal fusion peptides [58,59,60,61,62,63,64] and regions of high structural plasticity, e.g., the hinge region of fusion protein HIV-1 gp41 [65]. In fusion proteins such as influenza hemagglutinin [66,67], vesicular stomatitis virus (VSV) G-protein [68] and presynaptic SNAREs [69], the TM domain may translate a conformational change in the extramembrane domain into local bilayer stress [70,71], and fusogenicity of TM domains in SNARE or VSV G-protein is inversely correlated to its helicity in solution [72,73]. This is consistent with the presence in SNARE and other viral fusion protein TM domains [72,74] of β-sheet-promoting residues [75], *i.e.*, β-branched (I, V, T), glycine, or bulky (W, Y, F).

In both TM domain and C-terminal tail of CoV E proteins there is a high abundance of β-branched and bulky residues that can potentially destabilize α-helical conformation (Figure 1, red underlined). It has been proposed that E proteins may participate in inducing membrane curvature, or in the scission of particles [12,31,36,76,77,78]. Although fusion/fission activity of CoV E proteins has never been demonstrated, we hypothesize that this could be due to a lack of suitable *in vitro* assays, e.g., involving both E and M proteins. Examination of these possibilities should be complemented with structural studies targeting both E and M proteins in lipid bilayers by solution and solid state NMR.

### 1.4. Protein-Protein Interactions

The interaction between CoV E and M proteins has long been reported to contribute to M localization and virion formation [23,79,80,81,82,83]. M protein is the most abundant protein component of the virion, and responsible for its shape [84]. In addition to its three predicted TM domains, it has a large C-terminal extramembrane domain (~120 residues) exposed to the cytoplasm or to the interior of the virion [85], which forms contacts with the C-terminal tail of the E protein [23,33,82,83]. These interactions take place at the ER-Golgi intermediate compartment (ERGIC), the budding compartment of the host cell. These contacts are likely to be important for particle assembly [86] since M-M interactions are major drivers of viral envelope formation [84].

ΔE mutants in MHV produced revertants with a partial duplication of the M gene. The newly created M protein lacked most of its C-terminal cytoplasmic tail [87]. These results suggested a common role of the “new M” and E proteins in “dispersing or de-aggregating” M during packaging [87]. Because structural data of E and M proteins are very limited, the precise mechanism by which this takes place is not known.

Recent yeast-two hybrid searches of interacting partners of SARS-CoV E using the C-terminal extramembrane domain as a bait have produced abundant data, although the significance of only a few of these binders has been explored and reported [88,89]. One of these binders is the protein associated with Lin Seven 1 (PALS1) [88], which is part of a complex that controls polarity and tight junction formation in epithelia. This interaction was found to involve PALS1 PDZ domain and the last four C-terminal residues of SARS-CoV E protein, through a X-ϕ-X-ϕ motif, where ϕ is a hydrophobic amino acid. E protein hijacked PALS1 to the ERGIC and Golgi region, which was consistent with observed alterations of lung epithelia integrity. PDZ [post synaptic density protein (PSD95), *Drosophila* disc large tumor suppressor (Dlg1), and *zonula occludens*-1 protein (zo-1)] domains are common structural domains of 80–90 amino acids found in any organism. PDZ domains bind C-terminal tails of proteins, although internal binding sites have also been reported (reviewed in [90]). More than 150 PDZ domain-containing proteins with over 250 non-redundant PDZ domains have been recognized in the human proteome [91], and these are abundantly represented in protein–protein interactions that alter cellular pathways. The latter suggests a wider implication of this newly found interaction, as viruses could harness the alteration of these pathways to their own advantage.

In our model (Figure 3) of SARS-CoV E, the last C-terminal residues which include the proposed PDZ-binding motif [88,89] were truncated. However, in the same SDS/DPC micellar system [45], the secondary structure of the full length protein could be estimated using ^13^Cα chemical shifts. This indicated a predominant random coil structure in that part of the protein. In addition, a synthetic peptide encompassing the last 18 residues (59 to 76) also adopted a random coil conformation, whether in solution or in presence of lipid zwitterionic membranes [55]. This is somewhat surprising, since PDZ-binding motifs adopt typically a β-strand conformation [90]. However, the latter may be induced by binding. Alternatively, the conformation of this domain in the context of the full length protein and in presence of lipid membranes may be different from the one observed in micelles.

In addition to binding to C-terminal peptides, some PDZ domains recognize internal peptide fragments [92,93,94], e.g., Par (partitioning defective)-6 PDZ domain can bind to an internal peptide fragment from PALS1 that adopts an extended conformation [92]. Another example is the interaction between the PDZ domains from β-syntrophin or PSD-95 and the internal β-hairpin finger of the nNOS PDZ domain [93]. Therefore, the PDZ-binding domains of E proteins may not be restricted to its few C-terminal amino acids.

This C-terminal PDZ-binding motif found in SARS-CoV E protein was later shown to be a determinant of virulence, as infection of mice with viruses lacking this domain lowered their immune response [89]. In the latter paper, another yeast-two hybrid campaign using the same C-terminal SARS-CoV E domain discovered another binder, syntenin. The latter is a scaffolding protein that can initiate a signaling cascade resulting in the phosphorylation and activation of p38 mitogen-activated protein kinase (p38-MAPK) [95], leading to expression of proinflammatory cytokines. Disruption of this pathway may have clear therapeutic implications since in SARS-CoV-infected patients it is an exacerbated inflammatory response that leads to epithelial and endothelial damage, edema and acute respiratory distress syndrome (ARDS) [96]. However, a 12-residue deletion at the C-terminus resulted in a virus with high pathogenicity [33]. The reason for this discrepancy is not clear, but it suggests compensatory mechanisms or unpredictable effects of truncation length in local E protein structure. Thus, epithelial integrity and inflammatory response appear to involve this PDZ-binding domain. Noticeably, several other viruses, e.g., human papillomavirus and influenza A, have been found to enhance pathogenesis through proteins containing PDZ binding motifs (reviewed in [97]), which suggests that this is a particular case of a widely used viral strategy.

CoV E proteins may also interact with endogenous channels, modulating their function to the advantage of the virus. In *Xenopus* oocytes, it has been shown that co-expression of SARS CoV E with human epithelial sodium transporter (ENaC) decreased amiloride-sensitive current through activation of PKC and subsequent decrease in ENaC surface levels [98]. A similar direct or indirect inhibitory effect on other endogenous channels was proposed from patch clamp results in SARS-CoV E-transfected cells [26]. For IBV E, interaction with endogenous channels or SNAREs have been hypothesized to explain the rearrangement of the Golgi complex in response to expression of IBV E [36], although the involvement of the IBV E channel itself cannot be ruled out. For example, ion homeostasis at the Golgi could affect Na^+^/H^+^ exchangers that are critical for maintaining low luminal pH. Interactions of viroporins with Golgi channels or transporters are largely unexplored in the viroporins field, but notable cases have been already reported. For example, oncogenic protein E5 from papillomavirus [99] is able to bind the V_o_ subunit of the lumen-acidifying V-ATPase [100], preventing assembly of the pump and leading to alkalinization of the Golgi lumen [101].

## 2. The Small Hydrophobic (SH) Protein in the Respiratory Syncytial Virus (RSV)

### 2.1. The Respiratory Syncytial Virus

Human respiratory syncytial virus (hRSV) is an enveloped pneumovirus in the *Paramyxoviridae* family. hRSV was first isolated in 1956 from a chimpanzee with a respiratory illness, and later found to be a human virus [102]. hRSV is the leading cause of bronchiolitis and pneumonia in infants and elderly [103], and the most frequent cause of hospitalization of infants and young children in industrialized countries. In the general population, hospitalization rates are similar to those found for influenza infections [104].

In developing countries, RSV is a significant cause of death, with global estimates of more than 70,000 deaths in young children. hRSV is the third most important cause of deadly childhood pneumonia after *Streptococcus pneumoniae* and *Haemophilus influenzae* [105]. The epithelial cells of the respiratory tract are the major sites of virus replication, but hRSV can infect a wide variety of human and animal cells.

The fusion (F) protein facilitates viral entry through the cell membrane [106] through formation of a 6-helix bundle. This critical step has been targeted *in vitro*, e.g., peptides that mimic conserved domains of RSV-F protein [107,108], peptides based on F-interacting RhoA GTPase [109], dendrimer-like molecule RFI-641 [110], or other organic compounds [111,112]. Other approaches have involved gene transfer to expose viral proteins to cells [113], or siRNA against specific viral proteins [114]. Vaccines have been recently designed based on a stabilized RSV-F form which preserves a highly antigenic site in its prefusion state, yielding RSV-specific neutralizing antibodies in mice and macaques [115,116]. Recently, a vaccine candidate based on the extracellular domain (C-terminal) of RSV viroporin, the small hydrophobic (SH) protein, has been reported [117], and prevention of nasopulmonary infection in mice caused by RSV has been reported using stapled peptides targeting the fusogenic F-protein 6-helix bundle [118]. However, despite all these efforts, new FDA-approved drugs have yet to emerge.

Palivizumab is a humanized monoclonal antibody (IgG) directed against F protein but is only moderately effective [119], which combined with its high cost [120] limits its use to a small fraction of patients worldwide. The only licensed drug for use in infected individuals is ribavirin, a nucleoside analog, but its efficacy is very limited [121]. Naturally acquired immunity to RSV is neither complete nor durable, and recurrent infections occur frequently during the first three years of life. Therefore, low immunoprotection and lack of suitable antivirals makes imperative the search of new drug targets and strategies for effective treatment. The hRSV genome transcribes 11 proteins [122], including the three membrane proteins: fusion (F), small hydrophobic (SH), and attachment (G), which plays a role in the initial interaction of the virus with the cell [123,124].

### 2.2. The Small Hydrophobic Protein

The small hydrophobic (SH) protein is 64 (RSV subgroup A) or 65 (RSV subgroup B) amino acids long, with a single α-helical TM domain [125,126]. Both A and B subgroups are capable of inducing severe lower respiratory tract disease in humans [127,128,129]. Most SH protein accumulates at the membranes of the Golgi complex in infected cells, but it has also been detected in the endoplasmic reticulum and plasma membranes [130].

RSV that lacks SH (RSVΔSH) is still viable, and still forms syncytia [131,132,133], but it is attenuated *in vivo.* In mouse, RSV ΔSH replicated 10-fold less efficiently in the upper respiratory tract [132], whereas chimpanzees developed significantly less rhinorrhea than those infected with wild-type RSV [134]. Other reports have shown that lack of SH protein leads to an attenuated phenotype in children and in rats [133,135]. Overall, these results indicate involvement of hRSV SH protein in replication and pathogenesis. In fact, a recombinant RSV with deletion of the SH gene has been proposed as a live vaccine in calves [136] and in humans [133].

In common to SARS-CoV E protein, SH protein blocks or delays apoptosis in infected cells [137], and a similar anti-apoptotic effect of SH protein has been observed for other members of the *Paramyxoviridae* family that encode SH proteins, e.g., J Paramyxovirus (JPV) [138,139], mumps virus (MuV), and the parainfluenza virus 5 (PIV5), formerly known as simian virus 5 (SV5)–see below. In all these systems, SH protein seems to block apoptosis during infection through inhibition of the TNF-α pathway [137,138,140,141]. For example, apoptosis induced by PIV5 ΔSH was blocked by neutralizing antibodies against TNF-α and TNF-α receptor 1 (TNF-R1), but not by an antibody against TNF-R2 [140]. In addition, when the SH protein gene of PIV5 was substituted by SH protein from MuV or RSV (A2 or B1 strains) [137,141], apoptosis was prevented through blockage of the TNFα-mediated NF-κB pathway, suggesting that these SH proteins target similar pathways but through unknown mechanisms. By delaying apoptosis, the virus may evade the premature death of host cells, allowing viral replication.

Viruses are able to activate the inflammasome, which activates caspase-1 to produce pro-inflammatory cytokines, e.g., IL-1β. They do so by disrupting ion homeostasis through the expression of viroporins, which allow ion leakage from intracellular organelles into the cytosol. For example, influenza A virus (IAV) activates NLRP3 as a result of H^+^ or ion flux from Golgi mediated by the M2 ion channel [142]. The 2B protein from several picornaviruses, including the encephalomyocarditis virus (EMCV), poliovirus and enterovirus 71 were shown to induce NLRP3 cytoplasmic relocalization and inflammasome activation in an intracellular Ca^2+^-mediated manner [143]. The channel activity of SARS-CoV E protein is required for the processing of IL-1β [34], which requires caspase-1 activation. Channel activity of SH protein may also result directly or indirectly in activation of the NLRP3 inflammasome. Results consistent with this have been reported [144], where hRSV infection led to cleavage of pro-inflammatory cytokines, producing IL-1β and causing lung pathology and disease exacerbation. Further, an hRSV ΔSH mutant led to reduced IL-1β secretion and caspase-1 expression, whereas lipid raft disruptors, which may affect SH protein targeting to Golgi lipid rafts, blocked inflammasome activation [145]. Similar effects were observed by blocking SH protein channel activity [145]. However, the drugs used were not specific or particularly effective. Therefore, although a suggestion has been made to link inflammasome activation to SH protein channel activity and ion leakage from the Golgi during infection [145], this has not been confirmed.

### 2.3. Structural Studies and Relevant Domains of RSV SH Protein

**Figure 4:**
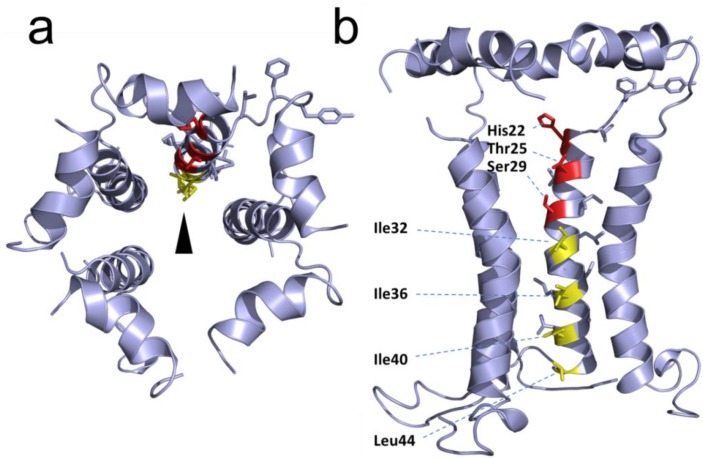
Structural model of RSV SH protein. Top view (**a**) and side view (**b**) of the SH protein pentamer [148]. In the latter, one monomer has been removed. The lumenally oriented polar residues (red) and hydrophobic residues (yellow) in the α-helical bundle are indicated.

The topology of RSV SH protein is just opposite to that of CoV E proteins, with N- and C-terminal extramembrane domains oriented cytoplasmically and lumenally/extracellularly, respectively. The mutual orientation of the transmembrane (TM) α-helices that form the ion channel was determined in lipid bilayers using site specific infrared dichroism [146,147]. A description of the full length SH protein monomer has been obtained by solution NMR in dodecylphosphocholine (DPC) micelles [126] and in bicelles [148]. Like SARS-CoV E, SH protein forms homopentameric channels [126,146] that have low ion selectivity [148]. The TM domain of SH protein has a funnel-like architecture [126] (Figure 4), as observed in other viroporins, e.g., influenza M2 [149], SARS E protein [46] and HCV p7 [150]. A narrower region [126] in the TM domain is lined with hydrophobic side chains (Ile32, Ile36, Ile40 and Leu44) whereas the more open N-terminal region is lined by polar residues, *i.e.*, His22, Thr25 and Ser29 (Figure 4b).

In RSV SH protein, the TM α-helix extends up to His-51 in the C-terminal region, followed by a loop, whereas the N-terminal cytoplasmic extramembrane domain forms a short α-helix (residues 5–14) (Figure 4b), which is present both in micelles and in bicelles [148].

**Figure 5:**
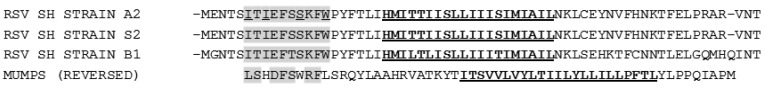
Alignment between SH protein for strains A2 and S2 (group A), B1 (group B) and MuV. Note that MuV SH is a type I integral membrane protein, *i.e.*, its C terminus faces the cytoplasm [154,155], therefore its orientation has been reversed for the alignment. The TM domain is underlined and bold, and a “10-residue” conserved fragment of high similarity has been highlighted in grey.

Several indications suggest the involvement of this exposed cytoplasmic (N-terminal) tail in the observed anti-apoptotic effects of SH protein (see above). For example, addition of a hemagglutinin antigen epitope tag at the cytoplasmic tail of PIV5 SH protein abolished its ability to inhibit TNF-α signaling [141]. Second, while the SH proteins of RSV and MuV (both human pathogens) have opposite topologies, they share a high degree of similarity in a 10-residue stretch at the cytoplasmic domain (N-terminus for RSV SH and C-terminus for MuV SH) (Figure 5). Third, SH proteins in MuV, PIV5 and JPV have extremely short lumenal domains (9, 2 and 10 residues, respectively) compared with their much longer cytoplasmic domains. Finally, hRSV SH protein sequence is most conserved at the N-terminal cytoplasmic domain [151,152,153].

### 2.4. Protein-Protein Interactions Involving hRSV SH Protein

The interaction between RSV SH and G proteins has been reported previously in infected cells [156,157], although its significance is not yet clear. F protein seems to be the main determinant of host cell specificity during virus entry [158], and both F and G proteins are able to bind heparin sulfate, the putative cell receptor for RSV [159]. However, a tri-component complex between SH, G and F proteins was not observed [156]. Both G and F proteins have one predicted TM domain, and interaction with SH protein can be both through the TM domain or extramembrane domains of the latter.

**Figure 6:**
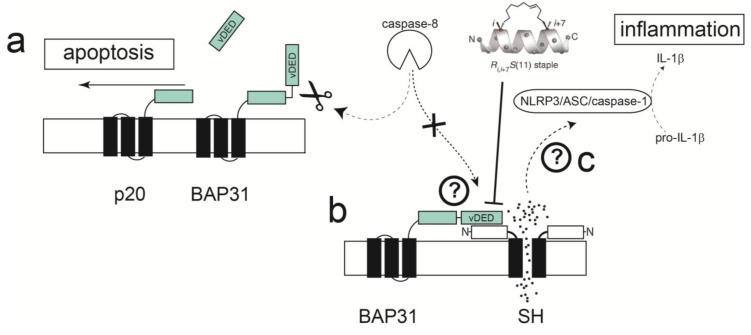
Schematic depicting a hypothetical model for apoptosis delay through interaction of SH and BAP31 proteins. (**a**) Activation of caspase 8 via TNF-α or other effectors cleaves the cytoplasmic vDED domain of BAP31, producing pro-apoptotic p20; (**b**) SH protein may bind to various proteins in this pathway, e.g., BAP31, blocking its cleavage by caspase-8; (**c**) ion channel activity through SH protein may contribute to production of IL-1β through activation of the inflammasome.

Recently, a membrane-based yeast two-hybrid system (MbY2H) was used to identify a cellular binding partner of hRSV SH protein, the B-Cell receptor-associated protein 31 (BAP31) [160], in a human lung cDNA library. BAP31 is a membrane protein located at the endoplasmic reticulum (ER) and has an essential role in sorting newly synthesized membrane proteins [161]. Additionally, BAP31 has a cytoplasmic C-terminus that form two coiled coils [162,163], one of them containing a variant of the death effector domain (vDED) [164] flanked by two caspase-8 cleavage sites. This domain is excised upon activation of caspase-8 [165,166] to produce a fragment p20, known to function as a proapoptotic factor [166,167]. BAP31 also forms a complex with the mitochondrial fission 1 (Fis1) [168] membrane protein, which spans the ER and mitochondria, and serves as a platform for activation of caspase-8. The consequences, or biological relevance, of the interaction between SH and BAP31 proteins are not known. These contacts may interfere between BAP31 and caspase-8 interaction, e.g., by SH protein binding to BAP31 vDED domain. This could in turn prevent the cleavage of BAP31 and the formation of pro-apoptotic p20, thus delaying apoptosis (Figure 6).

**Figure 7:**
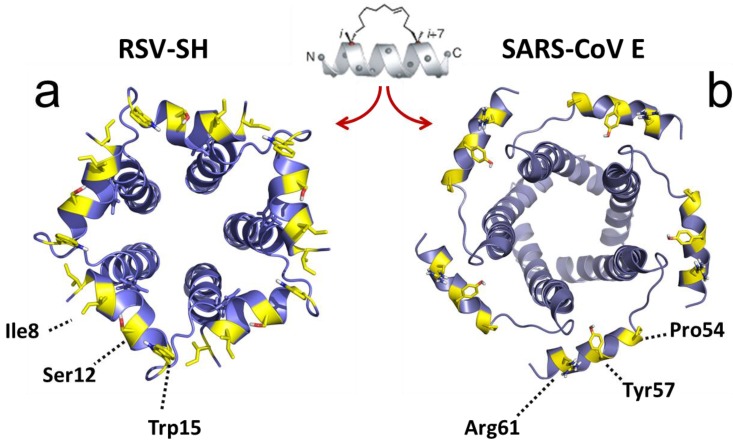
RSV SH and SARS-CoV E protein channels. (**a**) RSV SH protein channel and (**b**) SARS-CoV E protein channel, viewed from the cytoplasmic side [126,148]. The residues exposed to the cytoplasmic side (yellow) may be actively involved in protein-protein interactions and these cytoplasmic α-helices can be used as templates for stapled α-helical peptides.

The screening for SH protein binders referred to above [160] used a truncated form of SH protein, encompassing only the TM domain and the cytoplasmic domain (M1-L44), therefore only these domains are involved in interaction with BAP31. In the latter report, a stretch of residues in the N-terminal cytoplasmic helix of SH protein was perturbed by addition of the BAP31 vDED domain to full length SH protein in micelles, with major shifts observed at residues Ile6, Ile8, Ser12 and Trp15 (Figure 7a). Although there is conservation in that region between hRSV SH and mumps virus SH protein (Figure 5), the residues indicated above are not conserved, whereas PIV5 SH protein does not show any homology in this region, suggesting that the latter do not bind BAP31, or they do but through a different binding site.

A system with intriguing similarities to RSV SH protein is found in the human papillomavirus type 16 (HPV-16). Indeed, apoptosis induced by Fas ligand (FasL) or by tumor necrosis factor-related apoptosis-inducing ligand (TRAIL) was strongly suppressed in keratinocytes expressing E5 [169], a viroporin also found to bind BAP31 [170]. E5 impaired formation of the death-inducing signaling complex triggered by TRAIL, showing that inhibition of ligand-mediated apoptosis in human keratinocytes is a primary function of the HPV-16 E5 protein.

The mechanism of inhibition of TNF-α signaling by SH proteins is not clear, but a model has been proposed [141] where SH protein interacts directly or indirectly with TNF-R1, blocks TNF-α signaling, and prevents more TNF-α from being produced. To elucidate this mechanism it is very important to identify other RSV SH-interacting proteins that may be involved in apoptotic pathways. In addition, to unequivocally link SH protein ion channel activity to inflammasome activation, channel inactive mutants need to be tested. Obvious candidates are polar residues that face the lumen of the channel (Figure 4b), e.g., mutations H22A, T25A and S29A, and mutants that disrupt channel structure, *i.e.*, obtained by introducing bulky side chains in residues involved in TM helix-helix interactions.

### 2.5. Disruption of Protein-Protein Interactions; Potential for Clinically Relevant Strategies

Despite the fact that the precise mechanisms that link the viroporins E and SH with the delaying of apoptosis or triggering the inflammasome are not known, it is clear that novel strategies to combat viral infection can already be hypothesized.

For example, if the model shown in Figure 6 is confirmed, one can envisage the possibility that stapled peptides mimicking the α-helical cytoplasmic domain of SH protein can disrupt the interactions between SH-vDED, leading to enhanced apoptosis and cell death. Stapled α-helical peptides have two special amino acids bearing an olefinic side chain [171]. Prior to the final FMOC-deprotection step, the peptide-resin is subjected to ring-closing olefin metathesis (*i.e.*, stapling) which increases peptide stability and helicity. In principle, both membrane and extramembrane parts of proteins can be targeted, as shown recently for *Halobacterium salinarium* where efflux by a small multidrug resistance protein was inhibited with peptides targeting its TM domain [172]. In this case, peptides that disrupt monomer-monomer interaction in viroporins, and hence abolish channel activity, are still unexplored.

Once defined the interactions between the SARS-CoV E protein C-terminal tail and other cellular or viral binders, e.g., between E and M, a similar strategy could be followed by designing α-helical peptides that mimic the cytoplasmically exposed α-helical domain in the C-terminal tail of CoV E proteins (Figure 7b).

To conclude, both SARS-CoV E and RSV SH viroporins delay apoptosis in infected cells, which may help evade host inflammatory responses and the premature death of the host cells. This effect may be related to a common cytoplasmically oriented membrane-bound α-helix, connected to the TM domain by a flexible loop. It is becoming apparent that these cytoplasmically oriented α-helices can become templates for the design of stapled α-helical peptides that would disrupt protein-protein interactions with viral and host proteins, and these interactions potentially constitute novel drug targets to combat viral infection. However, more detailed mechanistic information should be available before these strategies can be widely used.
